# Completeness and timeliness of tuberculosis notification in Taiwan

**DOI:** 10.1186/1471-2458-11-915

**Published:** 2011-12-12

**Authors:** Hsiu-Yun Lo, Shiang-Lin Yang, Pesus Chou, Jen-Hsiang Chuang, Chen-Yuan Chiang

**Affiliations:** 1 Institute of Public Health, Community Medicine Research Center, National Yang-Ming University, Linong Street, Taipei 11221, Taiwan; 2 Department of Health, Centers for Disease Control, Linsen S. Road, Taipei 11050, Taiwan; 3 Department of Lung Health and NCDs, International Union Against Tuberculosis and Lung Disease, boulevard Saint-Michel, 75006 Paris, France; 4 Department of Internal Medicine, Wan Fang Hospital, Taipei Medical University, No 111, Section 3 Hsin-Long Road, Taipei 116, Taiwan; 5 Department of Internal Medicine, School of Medicine College of Medicine, Taipei Medical University, Wu-Hsing Street, Taipei 110, Taiwan

**Keywords:** Completeness, Notification, Reporting, Tuberculosis

## Abstract

Tuberculosis (TB) is a notifiable disease by the Communicable Disease Control Law in Taiwan. Several measures have been undertaken to improve reporting of TB but the completeness and timeliness of TB notification in Taiwan has not yet been systemically evaluated.

**Methods:**

To assess completeness and timeliness of TB notification, potential TB cases diagnosed by health care facilities in the year 2005-2007 were identified using the reimbursement database of national health insurance (NHI), which has 99% population coverage in Taiwan. Potential TB patients required notification were defined as those who have TB-related ICD-9 codes (010-018) in the NHI reimbursement database in 2005-2007, who were not diagnosed with TB in previous year, and who have been prescribed with 2 or more types of anti-TB drugs. Each potential TB case was matched to the national TB registry maintained at Taiwan Centers for Disease Control (CDC) by using national identity number or, if non-citizen, passport number to determine whether the patients had been notified to local public health authorities and Taiwan CDC. The difference in the number of days between date of anti-tuberculosis treatment and date of notification was calculated to determine the timeliness of TB reporting.

**Results:**

Of the 57,405 TB patients who were prescribed with 2 or more anti-tuberculosis drugs, 55,291 (96.3%) were notified to National TB Registry and 2,114 (3.7%) were not. Of the 55,291 notified cases, 45,250 (81.8%) were notified within 7 days of anti-tuberculosis treatment (timely reporting) and 10,041(18.2%) after 7 days (delayed reporting). Factors significantly associated with failure of notification are younger age, previously notified cases, foreigner, those who visited clinics and those who visited health care facilities only once or twice in 6 months.

**Conclusion:**

A small proportion of TB cases were not notified and a substantial proportion of notified TB cases had delayed reporting, findings with implication for strengthening surveillance of tuberculosis in Taiwan. Countries where the completeness and timeliness of TB notification has not yet been evaluated should take similar action to strengthen surveillance of TB.

## Background

Tuberculosis (TB) remains a major public health problem in Taiwan. In 2009, a total of 13827 TB cases (13336 new and 491 relapse) were notified to Taiwan Centers for Disease Control (CDC); the notification rate of TB was 59.9 per 100000 population and TB mortality was 3.2 per 100000 population [1]. Complete and timely notification of TB to public health authorities is one of the essential components of TB control [2]. In Taiwan, notification of TB initiates assessment of TB cases by public health nurses, assignment of directly observed therapy (DOT) observers, and contact tracing. Proper notification of TB provides surveillance data to keep track of the epidemic of tuberculosis.

TB is a notifiable disease by the Communicable Disease Control Law in Taiwan. Several measures have been undertaken to improve notification of TB. The Bureau of National Health Insurance (NHI) introduced the no-notification-no- reimbursement (NNNR) policy and the notification-fee (NF) policy in 1997, resulting in a prompt increase in TB notification and the number of notified TB cases reached a historical peak in 1997 [3]. Further, Taiwan CDC periodically cross matched vital registration and National TB registry to identify failure of notification of cases who died of TB.

The completeness and timeliness of TB notification in Taiwan has not yet been systemically evaluated. The Bureau of NHI maintains a reimbursement database containing health care services provided by health care facilities who have signed contracts with the Bureau of NHI. We use the NHI reimbursement database to assess completeness and timeliness of TB notification in Taiwan. We report results of this study.

## Materials

We identify potential TB cases diagnosed by health care facilities in 2005-2007 using prescription records of the NHI reimbursement database. Each potential TB case was matched to the national TB registry maintained at Taiwan CDC by using national identity number or, if non-citizen, passport number to determine whether the patient had been notified to local public health authorities and Taiwan CDC. The difference in the number of days between date of anti-TB treatment and date of notification was calculated to determine the timeliness of TB reporting.

### National health insurance database

The NHI Program was run by the government under the principle of mandatory and universal enrollment. The proportion of the population insured under the NHI Program was initially 92% when the NHI program was launched in 1995, and 99% of the populations was covered by NHI in the end of 2008 [4,5]. With the implementation of the NHI program, most hospitals and clinics (more than 90%) have signed contracts with the Bureau of NHI, and fee-for-service is reimbursed by the Bureau of NHI on a monthly schedule. The NHI reimbursement database is case-based, including individual information of national identity number, sex, birthday, dates of outpatient visit and hospitalization, examinations, diagnosis using the International Classification of Disease, ninth revision (ICD-9) coding system and treatment.

### TB services in Taiwan

TB service was mainly provided through a vertical programme in the past. Re-structuring of the TB control programme took place in 2001. Thereafter, diagnosis and treatment of TB have been provided through general health care system. Currently, TB patients can receive anti-TB treatment in any clinic or hospital, through the coverage of the NHI Program. However, there is no TB case register at facility level. Management of TB cases (DOT, contact tracing) are the responsibility of the public sector. The link between general health care facilities and the public sector for the management of TB cases is established through TB notification.

### TB notification and the computerized TB registration system

Taiwan has established a TB registry for decades. Taiwanese regulations stipulate that all suspected and confirmed TB cases should be reported to the local Department of Health in each city/county within 7 days. Upon receiving notification, a public health nurse (PHN) in each township/village health station is responsible for patients visit (within seven days of notification), completion of a standard registration form, and supervision of DOT observers in performing DOT. A computerized National TB Register with a registration network was established in 1994 [3]. With the establishment of the computerized system, the PHN in each township/village health station is responsible for entering reports of suspected and confirmed TB cases (reported cases) into the National TB Register via the registration network. A web-based TB notification system was launched in 2002. The proportion of TB notified through the web-based notification system was 78.8% in 2005, 88.5% in 2006, and 94% in 2007. The TB registry is also a case-based system including patients' national identity number (or, if non-citizen, passport number), birthday, sex, bacteriological examination (smear for acid-fast stain and culture for *M. tuberculosis*), site of disease (pulmonary TB, extrapulmonary TB), date of initiation of anti-TB treatment, treatment regimen, and outcome of treatment.

### Definition of TB case

For the purpose of this study, TB patients required notification are those who have TB-related ICD-9 codes (010-018) in the NHI reimbursement database in 2005-2007, who were not diagnosed with TB in previous year, and who have been prescribed with 2 or more types of anti-TB drugs, including Isoniazid (H), Ethambutol (E), Rifampin (R), Pyrazinamide (Z), Prothionamide (PAS), Aminogylcoside (streptomycin, amikacin, and kanamycin) and Quinolone (ciprofloxacin, levofloxacin, ofloxacin and moxifloxacin), within 6 months.

Procedure of investigation

1. We identified patients who had TB-related ICD-9 codes in the NHI reimbursement database in 2005-2007.

2. We excluded those who already had TB-related ICD-9 codes in previous year.

3. We checked whether patients who were newly assigned with TB-related ICD-9 codes have been prescribed with anti-TB drugs.

4. Those who have been prescribed with 2 or more types of anti-TB drugs were matched to the national TB registry by using national identity number or, if non-citizen, passport number to determine whether they had been notified by 30 June 2008. Cases who could not be found at the national TB registry were classified as not notified. For those who have been notified previously and who have retreatment of TB in 2005-2007, we checked whether they were re-notified as retreatment cases. If not, they were also classified as not notified.

5. We investigated factors associated with failure of notification.

6. We calculated the difference in the number of days between date of initiation of anti-TB treatment and date of notification to assess timeliness of notification. Cases notified within 7 days of anti-TB treatment are classified as having timely reporting and after 7 days delayed reporting.

7. We investigated factors associated with delayed reporting.

8. We checked the completeness of data entry of critical fields of notification form, including name, sex, birthday, address, reporting health care facilities, medical chart number, name of reporting doctor, date of diagnosis, site of disease (pulmonary and/or extrapulmonary) and smear examinations.

### Statistics analysis

SAS 9.2 (SAS Institute Inc., Cary, NC, USA) and Microsoft Excel 2007 were used for data management and analysis. We assessed whether, age group, sex, type of case, nationality, type of health care facilities, and number of visit to health care facilities in 6 months were associated with failure of notification of TB and delayed reporting in univariate analysis by chi square test. Variables that were significantly associated with failure of notification or delayed reporting in a univariate analysis (*P *< 0.05) were selected for multivariable logistic regression analyses by stepwise approach and likelihood ratio test was used to determine the final fitted model. Stratified analysis was performed to assess interaction in under-notification between nationality, type of cases and number of health care facilities visits in 6 months, and in delayed reporting between age groups, type of cases and type of health care facilities. *P *value < 0.05 was regarded as statistically significant.

This study was approved by the Institute Review Board of Taiwan CDC.

## Results

**Figure 1:**
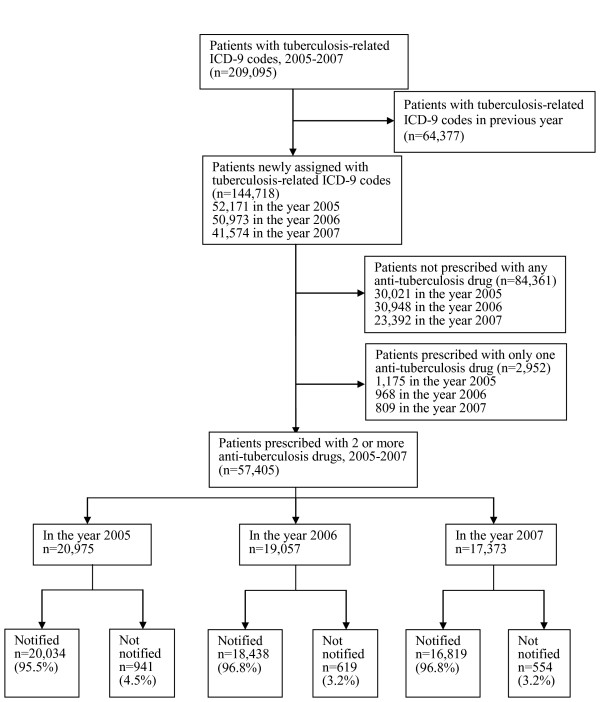
**Flow chart of ascertainment of notification of tuberculosis patients reimbursed by national health insurance in Taiwan, 2005-2007**.

There were 209,095 patients who had TB-related ICD-9 codes in the year 2005-2007 in the NHI reimbursement database. (Figure 1) Of the 209,095 patients, 64,377 (30.8%) had been assigned with TB-related ICD-9 codes in previous year and 144,718 (69.2%) were newly assigned with TB-related ICD-9 codes in the year 2005-2007. Of the 144,718 patients, 84,361 (58.3%) were not prescribed with any anti-TB drugs within 6 months after being assigned with TB-related ICD-9 codes, 2,952 (2.1%) were prescribed with one anti-TB drug, 57,405 (39.7%) were prescribed with 2 or more type of anti-TB drugs. Of the 57,405 patients, 55,291 (96.3%) were notified and 2,114 (3.7%) were not. Of the 57,405 patients, 52,763 (91.9%) were new TB cases and 4,642 (8.1%) were retreatment cases who had been notified previously. Of the 52,763 new TB cases, 1,270 (2.4%) were not notified, and of the 4,642 retreatment cases, 844 (18.2%) were not notified. (*p *< 0.001)

Table 1 shows factors associated with failure of notification of TB. The proportion of un-notified cases was higher among patients aged 25-44 years (5.6%) (*p *< 0.001), retreatment cases (18.2%) (*p *< 0.001), foreigners (22.8%) (*p *< 0.001), those who visited clinics (5.9%) (*p *< 0.001), and those who visited health care facilities only once (14.1%), or twice (8.2%) (*p *< 0.001), as compared with other groups. In multivariate analysis, factors significantly associated with failure of notification of TB were age groups (≦ 24 years, adjOR 1.8, 95% CI 1.5-2.3; 25-44 years, adjOR 2.1, 95% CI 1.8-2.4; 45-64 years, adjOR 1.5, 95% CI 1.3-1.7; as compared with aged 65 years or more), retreatment cases (adjOR 11.5, 95% CI 10.4-12.8), foreigner (adjOR 6.8, 95% CI 5.6-8.1), type of health care facilities (clinic, adjOR 2.3, 95% CI 1.9-2.7; district hospital, adjOR 1.7, 95% CI 1.5-1.9; regional hospital, adjOR 1.2, 95% CI 1.1-1.3. as compared with medical centers) and number of visits to health care facilities in 6 months (once, adjOR 11.4, 95% CI 10.2-12.7; twice, adjOR 5.3, 95% CI 4.6-6.2, as compared with 3 times or more).

**Table 1:** Factors associated with failure of notification of tuberculosis in Taiwan, 2005-2007

	Total n = 5,7405 n (column%)		Notified n = 55,291 n (row%)		Not notified n = 2,114 n (row%)		Multivariate analysis adjOR (95% CI)	
Age group (y/r)*								

0-24	3,584	(6.2)	3,459	(96.5)	125	(3.5)	1.8	(1.5-2.3)

25-44	9,996	(17.4)	9,441	(94.4)	555	(5.6)	2.1	(1.8-2.4)

45-64	15,072	(26.3)	14,554	(96.6)	518	(3.4)	1.5	(1.3-1.7)

≥65	28,753	(50.1)	27,837	(96.8)	916	(3.2)	1.0	

Sex*								

male	38,534	(67.1)	37,180	(96.5)	1,354	(3.5)		

female	18,869	(32.9)	18,111	(96.0)	758	(4.0)		

Type of case*								

new cases	52,763	(91.9)	51,493	(97.6)	1,270	(2.4)	1.0	

previously notified cases	4,642	(8.1)	3,798	(81.8)	844	(18.2)	11.5	(10.4-12.8)

Nationality*								

citizen	56,024	(97.6)	54,225	(96.8)	1,799	(3.2)	1.0	

foreigner	1,381	(2.4)	1,066	(77.2)	315	(22.8)	6.8	(5.6-8.1)

Type of health care facilities*								

medical center	19,770	(34.4)	19,269	(97.5)	501	(2.5)	1.0	

regional hospital	22,664	(39.5)	21,854	(96.4)	810	(3.6)	1.2	(1.1-1.3)

district hospital	11,273	(19.6)	10,689	(94.8)	584	(5.2)	1.7	(1.5-1.9)

clinic	3,698	(6.4)	3,479	(94.1)	219	(5.9)	2.3	(1.9-2.7)

Number of visits to health care facilities in 6 months*								

once	7,876	(13.7)	6,763	(85.9)	1,113	(14.1)	11.4	(10.2-12.7)

twice	3,722	(6.5)	3,417	(91.8)	305	(8.2)	5.3	(4.6-6.2)

3 or more	45,807	(79.8)	45,111	(98.5)	696	(1.5)	1.0	

* Statistically significant in univariate analysis (*P *< 0.05).

Table 2 shows TB notification among citizen and foreigner, stratified by type of case and number of health facilities visits. The proportion of previously notified cases among citizens was higher than that among foreigners (8.3% vs. 0.5%). The interaction between nationality and type of case was statistically significant (*p *= 0.04). The association between type of case and under-notification was statistically significant among citizens but not among foreigners. Under-notification of new TB cases was substantial among foreigners but not among citizens (22.9% vs. 1.9%). The proportion of patients who had visited health facilities for only once in 6 months was higher among foreigners than that among citizens (31.1% vs. 13.3%). The interaction between nationality and number of visits to health care facility in 6 months was also statistically significant (nationality * once: *p *< 0.01; nationality * twice: *p *= 0.63). Under-notification of those who visited health facilities only once (43.7%) or twice (37.0%) was particularly high among foreigners.

**Table 2:** Tuberculosis notification among citizen and foreigner, stratified by type of case and number of health facilities visits

	Total n (column %)		Not notified n (row%)		Notified n (row %)		adjOR*	(95% CI)	p
Citizen									

previously notified cases	4,635	(8.3)	843	(18.2)	3,792	(81.8)	11.7	(10.5-13.1)	< 0.001

new cases	51,389	(91.7)	956	(1.9)	50,433	(98.1)			

Foreigner									

previously notified cases	7	(0.5)	1	(14.3)	6	(85.7)	1.9	(0.1-10.7)	0.88

new cases	1,374	(99.5)	314	(22.9)	1,060	(77.1)			

Citizen									

health facilities visits only once	7,446	(13.3)	925	(12.4)	6,521	(87.6)	12.0	(10.7-13.5)	< 0.001

health facilities visits twice	3,576	(19.1)	251	(7.0)	3,325	(93.0)	5.2	(4.4-6.1)	< 0.001

health facilities visits ≥3 times	45,002	(80.3)	623	(1.4)	44,379	(98.6)	ref.		

Foreigner									

health facilities visits once	430	(31.1)	188	(43.7)	242	(56.3)	7.6	(5.6-10.4)	< 0.001

health facilities visits twice	146	(10.6)	54	(37.0)	92	(63.0)	5.8	(3.8-8.8)	< 0.001

health facilities visits ≥3 times	805	(58.3)	73	(9.1)	732	(90.9)	ref.		

* Adjusted by age, type of health care facilities and type of cases or number of health facilities visits

Of the 55,291 notified cases, 45,250 (81.8%) were notified within 7 days of anti-TB treatment; 10,041(18.2%) were notified after 7 days (14.5% in 8-30 days, 2.9% in 31-90 days and 0.7% after 90 days) and were considered as having delayed reporting. Higher proportions of new cases were notified within 7 days of anti-TB treatment (81.3% in 2005, 82.8% in 2006 and 84.1% in 2007) as compared with retreatment cases (73.5% in 2005, 72.8% in 2006, 65.9% in 2007) (*p *< 0.001). (Table 3). The proportion of retreatment cases with timely reporting was 73.5% in 2005, which decreased to 65.9% in 2007.

**Table 3:** Timeliness of notification of tuberculosis in Taiwan, 2005-2007

	Number of patients (%)		New cases						Previously notified cases					
			
			2005		2006		2007		2005		2006		2007	
			
			n	(%)	n	(%)	n	(%)	n	(%)	n	(%)	n	(%)
Total	55,291	100.0	18,662	100.0	17,135	100.0	15,696	100.0	1,372	100.0	1,303	100.0	1,123	100.0

0-7 days	45,250	81.8	15,176	81.3	14,181	82.8	13,196	84.1	1,008	73.5	949	72.8	740	65.9

8-30 days	8,006	14.5	2,771	14.8	2,362	13.8	2,040	13.0	280	20.4	273	21.0	280	24.9

31-90 days	1,630	2.9	555	3.0	477	2.8	399	2.5	51	3.7	62	4.8	86	7.7

≧ 91 days	405	0.7	160	0.9	115	0.7	61	0.4	33	2.4	19	1.5	17	1.5

Table 4 shows factors associated with delayed reporting. In multivariate analysis, factors significantly associated with delayed reporting were the following: patients aged 45-64 years (adjOR 1.2, 95% CI 1.1-1.3), ≧ 65 years (adjOR 2.0, 95% CI 1.9-2.1), as compared with patients aged ≦ 24 years; previously treated cases (adjOR 1.9, 95% CI 1.8-2.1) as compared with new cases; citizens (adjOR 1.8, 95% CI 1.4-2.2) as compared with foreigners; patients treated in medical center (adjOR 3.8, 95% CI 3.3-4.4), regional hospital (adjOR 2.9, 95% CI 2.5-3.4) and district hospital (adjOR 2.5, 95% CI 2.2-2.9) as compared with clinic patients; those who visited health care facilities once (adjOR 2.9, 95% CI 2.7-3.0), or twice (adjOR 1.6, 95% CI 1.5-1.7) in 6 months as compared with 3 times or more. The interaction between age group and type of health facility, and between type of case and type of health facility was statistically significant. Stratifying delayed notification among age groups and type of cases by types of health facilities demonstrated that the proportion with delayed notification was consistently highest in medically centers, followed by regional hospital, district hospital and clinics in all strata (data not shown).

**Table 4:** Factors associated with delayed reporting of tuberculosis in Taiwan, 2005-2007

	Total n = 55,291		Timely n = 45,250		Delayed n = 10,041		Multivariate analysis	
	**n (column%)**		**n (row%)**		**n (row%)**		**adjOR**	**(95% CI)**

Age group* (y/r)								

0-24	3,459	(6.3)	3,131	(90.5)	328	(9.5)	1.0	

25-44	9,441	(17.1)	8,381	(88.8)	1,060	(11.2)	0.9	(0.8-1.0)

45-64	14,554	(26.3)	12,412	(85.3)	2,142	(14.7)	1.2	(1.1-1.3)

≥65	27,837	(50.3)	21,326	(76.6)	6,511	(23.4)	2.0	(1.9-2.1)

Sex*								

female	18,111	(32.8)	14,957	(82.6)	3,154	(17.4)		

male	37,180	(67.2)	30,293	(81.5)	6,887	(18.5)		

Type of case*								

new cases	51,493	(93.1)	42,553	(82.6)	8,940	(17.4)	1.0	

previously notified cases	3,798	(6.9)	2,697	(71.0)	1,101	(29.0)	1.9	(1.8-2.1)

Nationality*								

foreigner	1,066	(1.9)	978	(91.7)	88	(8.3)	1.0	

citizen	54,225	(98.1)	44,272	(81.6)	9,953	(18.4)	1.8	(1.4-2.2)

Type of health care facilities*								

medical center	19,269	(34.9)	15,150	(78.6)	4,119	(21.4)	3.8	(3.3-4.4)

regional hospital	21,854	(39.5)	17,909	(81.9)	3,945	(18.1)	2.9	(2.5-3.4)

district hospital	10,689	(19.3)	8,934	(83.6)	1,755	(16.4)	2.5	(2.2-2.9)

clinic	3,479	(6.3)	3,257	(93.6)	222	(6.4)	1.0	

Number of visits to health care facilities in 6 months*								

1	6,763	(12.2)	4,303	(63.6)	2,460	(36.4)	2.9	(2.7-3.0)

2	3,417	(6.2)	2,613	(76.5)	804	(23.5)	1.6	(1.5-1.7)

≥3	45,111	(81.6)	38,334	(85.0)	6,777	(15.0)	1.0	

* Statistically significant in univariate analysis (*P *< 0.05).

Completion of data entry of critical fields of notification form were 100% for patients' name, sex, birthday, address, reporting health care facilities, medical chart number, name of reporting doctors, and date of diagnosis. Of the 55291 patients, 21 (0.04%) had no information on site of disease. Of the 51992 pulmonary TB cases, 1386 (2.7%) had no information on smear examinations.

## Discussion

Accurate, complete and timely reporting of notifiable diseases provide surveillance data to estimate disease burden, detect sporadic outbreaks and monitor epidemiological trends. Active surveillance may strengthen the completeness and accuracy of passive surveillance data [6-8]. As the majority of Taiwan citizens have been participated in the NHI program, the NHI reimbursement database offers a unique opportunity to conduct active surveillance to investigate completeness and timeliness of TB notification in Taiwan. We used prescription of 2 or more anti-TB drugs to identify potential TB cases diagnosed by clinicians. This approach has been applied in other settings previously [9,10]. Patients who were prescribed with only one anti-TB drugs was not included in this study because the majority of these cases were prescribed with either isoniazid, an aminoglycoside or a fluoroquinolone alone and may not fit the case definition of active TB.

Completeness of reporting of notifiable infectious diseases has been reported previously. Doyle and colleagues investigated reporting of notifiable infectious diseases in the US and found that reporting completeness varied from 9% to 99% and was most strongly associated with the disease being reported [6]. Studies in the US using various combinations of data of laboratory, clinic, pharmacy, and hospital discharge records reported that reporting completeness for TB ranged from 65% to 99.5% [8,10-13]. In United Kingdom, under-notification ranged from 7% to 27% [14,15]. Backer et al. listed several reasons related to under reporting, including insufficient consultation hours, patient privacy, not knowing notification is required, reporting severe disease only, lack of laboratory confirmation, considering other health care worker will report, and not knowing report procedure [16].

Under-reporting of TB has been an issue in private sectors as well as public sectors not involved in national TB programme. Chiang et al. did a survey on TB services in big cities and reported substantial under-reporting in Bangkok, Dhaka, Jakarta, Karachi, Kathmandu, and Manila [17]. Masjedi et al. investigated notification of 646 smear positive TB patients detected in the private laboratories in Tehran and reported that 87.3% of these patients could not be traced [18]. Improved TB notification by public-public and public-private mix approach has been reported from studies in China [19], India [20], Indonesia [21], Kenya [22], Myanmar [23], Nepal [24], and Vietnam [19]

Our study revealed that under-notification of TB in Taiwan was not high and decreased from 4.5% in 2005 to 3.2% in 2007, probably reflecting the impact of activities in strengthening surveillance of TB in Taiwan. However, timeliness of TB reporting was a concern because 18.8% of cases had delayed reporting. Younger age groups, especially those aged 25-44 years old, were more likely to be under reported. We speculated that TB may affect their job opportunity resulting in under reporting to keep TB confidential, which highlighted the importance of health education on TB in Taiwan. While delayed reporting decreased from 19.2% in 2005 to 17.1% in 2007 in the study population, delayed reporting occurred more frequently among the elderly. We speculated that this was due to difficulty in the diagnosis of TB among the elderly whose clinical manifestations were complicated by co-morbidity. Previously treated cases were substantially under notified (18.2%) and if notified, had delayed reporting (29.0%), probably because the notification system was not clear in guiding re-notification of patients who had already been registered previously. However, under-notification among foreigners was substantial not only in previously notified TB cases but also new TB cases. Further, a high proportion of foreigners have visited health care facilities for only once or twice in 6 months. We speculated that these were because they left Taiwan after the diagnosis of TB. Patients who visited health care facilities once or twice in 6 months were associated with under notification and delayed reporting. We did not know what happen to them (including foreigners). They might be died, or defaulted and remained source of transmission in the community.

Timeliness of notification of infectious disease and TB has been reported in other settings. Yoo et al. reported that time from disease onset to diagnosis contributed most to the delay in reporting [25]. Ward et al. reported that reporting of infectious diseases through an internet based reporting system had less reporting delay as compared with reporting through the conventional paper-based system [26]. Curtis et al. reported that the proportion of cases with delayed reporting (> 7 days) varied from 5% to 53% between sites and that factors associated with delayed reporting included infectiousness, type of provider, diagnosing provider, and reporting source [8]. Our study revealed that medical centers had the lowest proportion of patients under-notified but the highest proportion of patients with delayed reporting. We speculated that medical centers were more likely to have a system in place to ensure completeness of TB notification. Those who were not reported were captured at a later point in time and notified, and were classified as delayed reporting.

Completion of data entry of critical fields of notification form were 100% for patients' name, sex, birthday, address, reporting health care facilities, medical chart number, name of reporting doctors, and date of diagnosis, partly because these fields must be entered in the web-based notification system and because the majority were notified through the web-based system. Sputum smear examinations have been emphasized but were not set as "must enter" reporting fields in the web-based notification system to facilitate timely reporting. Clearly, it is essential to strengthen the information system to obtain results of smear examination of all pulmonary TB cases.

There were constraints of our study. First, results of bacteriological examinations were not available in the NHI reimbursement database. We were not able to determine whether under-notification was associated with bacteriological results. Second, prescription of 2 or more anti-TB drugs was used to identify TB cases. We may miss TB cases who were not prescribed with anti-TB drugs. Third, the NHI database did not specify history of TB treatment. Therefore, previously treated cases may be miss-classified as new cases. Fourth, TB cases who did not participate in the NHI program were not captured by this study. We may under-estimate under notification of these patients. Finally, we did not use capture-recapture technique to estimate completeness of reporting [27]. However, the number of cases included in this study was large and the proportion of population covered by the NHI program was very high, implying that findings of this study is relevant in strengthening surveillance of TB in Taiwan.

## Conclusion

In conclusion, a small proportion of TB cases prescribed with 2 or more anti-TB drugs were not notified and a substantial proportion of notified TB cases had delayed reporting. Under-notification of previously notified TB cases requires immediate attention of the surveillance system as the risk of drug-resistance among retreatment TB cases was substantially higher than that among new TB cases. Taiwan CDC should address factors associated with under-notification and delayed reporting to strengthen surveillance of TB in Taiwan. Countries where the completeness and timeliness of TB notification has not yet been evaluated should take similar action to strengthen surveillance of TB.

## Competing interests

The authors declare that they have no competing interests.

## Authors' contributions

HYL and CYC proposed and designed the protocol of the study; HYL and SLY performed data analysis and interpreted the results; HYL wrote the first draft of the manuscript; PC and JHC discussed and revised the first draft of manuscript. CYC critically revised and finalized the manuscript. All authors read and approved the final manuscript.

## Pre-publication history

The pre-publication history for this paper can be accessed here:

http://www.biomedcentral.com/1471-2458/11/915/prepub
